# Report From the Second Global Scientific Conference on Clinical Trial Design and Outcome Measures for *RDH12*-Associated Inherited Retinal Degeneration

**DOI:** 10.1167/tvst.13.8.17

**Published:** 2024-08-09

**Authors:** Silvia Cerolini, Jean Bennett, Bart P. Leroy, Todd Durham, Courtney Coates, Mathew T. Pletcher, Sue Lacey, Tomas S. Aleman

**Affiliations:** 1 Eyes on the Future, London, UK; 2 Scheie Eye Institute, Department of Ophthalmology, Perelman School of Medicine, University of Pennsylvania, Philadelphia, PA, USA; 3 Department of Ophthalmology and Center for Medical Genetics, Ghent University Hospital and Department of Head & Skin, Ghent University, Ghent, Belgium; 4 Foundation Fighting Blindness, Columbia, MD, USA; 5 Hope in Focus, Ledyard, CT, USA; 6 RDH12 Fund for Sight, Boiling Springs, SC, USA; 7 Astraea Medical Consulting, Hindhead, UK

**Keywords:** inherited retinal degeneration (IRD), RDH12, outcome measures, endpoints, clinical trial design

## Abstract

Following the success of the first multi-stakeholder workshop to accelerate the development of treatments for patients with *RDH12*-associated inherited retinal dystrophies in 2019, the patient organizations Eyes on the Future and the RDH12 Fund for Sight convened a second one day, multi-stakeholder workshop in November 2022, focusing on the challenges in clinical trial design and outcome measure selection for studies involving patients with *RDH12*-associated inherited retinal disease (IRD). Face to face and virtual participants included representatives from patient organizations, academic and industry clinicians, and scientists, as well as representatives from multiple regulatory agencies. Key areas of discussion focused on *RDH12* phenotypic heterogeneity, retinal structural-functional relationships, and the targeting of areas most likely to respond to gene therapies, the need for comprehensive natural history data to inform appropriate outcome measures, and the outcome measures most valued by the patient community including the lack of validated patient reported outcome measures. Learnings from recent IRD gene therapy trials and their relevance for *RDH12*-associated IRDs were discussed. Innovative trial designs with outcome measures selected relevant for the *RDH12* patient population, adaptive strategies, collaboration among scientists and clinicians, alignment among regulators, and close engagement with the patient community can enhance the future feasibility and success of clinical studies for *RDH12* retinal dystrophy.

**Translational Relevance:**

A multi-stakeholder, patient centric approach will be critical to the design of future successful clinical trials with outcome measures relevant to the *RDH12*-IRD population*.*

## Introduction

The Global Scientific Conference on Clinical Trial Design and Outcome Measures for Inherited Retinal Dystrophies Associated with Mutations in the *RDH12* Gene was hosted by the *RDH12* Alliance in partnership with Foundation Fighting Blindness and Hope in Focus and brought together key opinion leaders in the field of inherited retinal diseases (IRDs) from academic and clinical settings, regulatory agencies, biotechnology and pharmaceutical industry representatives, and patient organizations. The meeting was a follow-up to the first *Workshop on Accelerating the Development of Treatments for Inherited Retinal Dystrophies Associated with Mutations in the RDH12 Gene* in 2019.^1^

Recent clinical development challenges in retinal gene therapy, particularly those surrounding the selection of disease appropriate outcome measures in trial design, the acceptance by regulators of clinical trial endpoints beyond direct assessments of visual acuity, and the use of surrogate biomarkers of efficacy, prompted the convening of this second multi-stakeholder workshop, with the following goals:

• To understand patient expectations for meaningful treatment outcomes
• To gain insight into the most robust designs and clinically relevant outcome measurements for future *RDH12*-associated IRD clinical studies
• To identify the most significant challenges associated with development of therapies for *RDH12*-associated IRD and proposals for solutions
• To align on an action plan for further validation of meaningful endpoints

The format of the Workshop comprised didactic presentations from clinicians and the sharing of key insights from *RDH12* patient advocacy organizations, and clinical development learnings from industry participants. These served to inform the subsequent round table discussions on *RDH12* clinical study design and selection of appropriate endpoints. Presentations focused on latest learnings and expectations for treatment of *RDH12*-associated retinal degenerations, and on how lessons from the licensed ocular gene therapy, voretigene neparvovec-rzyl (LUXTURNA) and from recent IRD clinical studies may be translated to *RDH12*. This report focuses on the key discussion elements and actionable outcomes from the Workshop.

### Latest Understanding of *RDH12* Retinal Dystrophies

Bi-allelic pathogenic variants in the Retinol Dehydrogenase 12 gene (*RDH12*) cause extensive, early-onset, retina disease and comprise between 3% and 10% of cases of Leber congenital amaurosis (LCA), depending on the population studied.^2^^–^^5^ Although understanding of *RDH12*-associated retinal dystrophies is advancing, the exact mechanisms underlying the human disease are not fully understood, and the disease course and natural history are yet to be fully elucidated.

RDH12 is a member of a large group of dehydrogenases in the retina and is localized to the inner segments of the rod and cone photoreceptors. RDH12 has a role in the visual cycle and in maintaining rod and cone health.^6^^,^^7^ Along with RDH8, RDH12 provides most of the reductase activity within the inner and outer segments of photoreceptors which is required to reduce post-photoactivation all-trans-retinal to all-trans-retinol, a critical step for photoreceptor pigmentary regeneration.^8^ More importantly, RHD12 plays a protective role against retinaldehyde-induced cytotoxicity within photoreceptor cells.^8^^–^^10^

Clinical descriptors of the phenotype of patients with *RDH12* mutations include LCA, early onset retinal dystrophy (EORD), autosomal recessive retinitis pigmentosa, cone rod dystrophy, and late onset cone rod dystrophy.^11^ The variety of clinical names used to describe the disease inappropriately suggest phenotypic variability in *RDH12*-associated IRDs, which would pose a significant challenge to the understanding of the natural history of the disease, in predicting anticipated benefits from potential therapies, and in the selection of appropriate windows for treatment and outcome measures and endpoints for clinical studies. Careful review of the literature and recent detailed characterizations of the disease, however, paint a different picture, with common structural and functional denominators that are critical to both the understanding of the disease as well as to the potential for rescue by genetic treatments. Independent of the age at presentation of the recessive disease, the phenotypic expression includes a progressive, retina-wide pigmentary retinopathy with peripapillary sparing and prominent central changes that result in early impairment of the central visual function and thus visual acuity, and relative preservation of the peripheral rod, and, perhaps, cone function.^12^ Whereas photoreceptors and the retinal pigment epithelium (RPE) mainly within the central retina are severely degenerated, the nasal retina often reveals islands of preserved photoreceptor structure and function, even in the latter stages of the disease, suggesting these areas may be appropriate gene therapy targets.^5^^,^^12^^,^^13^ But the lack of consistency in accurately assigning these *RDH12*-associated phenotypes to the same diagnostic category does underscore the necessity of a genetic diagnosis.

When designing future *RDH12*-IRD clinical studies, an understanding of the retinal structural-functional relationship is critical, Tomas Aleman from the University of Pennsylvania (UPenn) emphasized. In *RPE65-*, *GUCY2D-*, or *CEP290*-IRDs, there is structural-functional dissociation, where despite patients experiencing often severe visual impairment, the integrity of retinal structures is still relatively maintained even in later disease stages.^14^^–^^16^ This pattern is the ideal scenario for gene therapies, as evidenced by the success of the gene augmentation clinical trials that led to the US Food and Drug Administration (FDA) approval of the first ocular gene therapy, voretigene neparvovec-rzyl (LUXTURNA) in 2017.^17^^,^^18^ Recent work at UPenn suggests *RDH12*-associated IRDs may show such a pattern of structural-functional dissociation in the better preserved regions, despite early and significant structural disorganization and associated functional loss. Additionally, whereas in some IRDs, despite photoreceptor degeneration, RPE may be spared at least temporally, *RDH12*-associated IRDs are characterized by both photoreceptor and RPE loss, often localized to specific areas of the retina, a situation that should be considered when selecting patients and areas amenable for treatment.^12^ Work supported by the RDH12 Fund for Sight is ongoing at UPenn to confirm these early observations.

Near infrared fundus autofluorescence (NIR FAF) examination of *RDH12* affected retinas (from pediatric to more advanced disease stages) show that despite structural abnormalities in the central retina, there can be preservation of the autofluorescent signal that originates from residual RPE underlying relatively preserved photoreceptors. Residual visual cycle activity can often be demonstrated by short-wavelength (SW) FAF in the foveal center and in areas of photoreceptor preservation in the peripapillary and nasal retina, in contrast to *RPE65*-IRDs where the visual cycle is compromised. Consistent with a central to mid-peripheral disease, optical coherence tomography (OCT) imaging reveals a very steep transition between the central areas of the retina and peripapillary areas, which are anatomically much closer to a healthy tissue. Research efforts conducted at UPenn to elucidate a correlation between structure and function in *RDH12* yielded several considerations. Two-color dark-adapted perimetry, full-field stimulus testing (FST) and pupillometry showed that both pediatric and adult *RDH12* patients have islands of preserved photoreceptors with relatively well-preserved rod and cone function near the central retina indicating that, although abnormal, the photoreceptors functionality could potentially be restored.^12^ The tests also revealed random pockets of relative preservation of the outer retina near the fovea, explaining the variation in visual acuity between eyes and among patients. These pockets may sustain functional vision, likely contributing to a variable age of symptomatic presentation which likely occurs when degeneration encroaches upon this critical area despite being present from much earlier in life.

Co-localized structural and functional comparisons using OCT and two-color dark-adapted perimetry showed cone sensitivity is nearly always severely abnormal but may be detectable near the foveal center, consistent with the abnormal visual acuities and severe foveal thinning characteristic of this disease. Surprisingly, rod function is also often detected within the pericentral retina and within relatively spared areas, and often in the more peripheral retina, as documented by FST and chromatic pupillometry. Of note, areas that appeared structurally healthy showed abnormal rod function indicating a potential dissociation between the retinal structure and the function of rods, and, perhaps, also cones. Interestingly, the structure/function relationship in *RDH12*-mediated IRD differs depending on the region of the retina that is analyzed, with the foveal area (mostly cones) showing no structural-functional dissociation, whereas in the pericentral area and peripapillary there can be localized structural-functional dissociation.

The findings suggest the topography of the structural-functional relationships should be considered when selecting retinal regions that may be targeted by new treatments, not only the regional topography of detectable photoreceptors, as for *RPE65*-IRD. Cross-sectional and longitudinal observations in patients with *RDH12*-IRD suggest there is a relatively asymptomatic period of fast degeneration of the pericentral retina within the first decade of life, that leads to a variable symptomatic presentation governed by the timing and severity of the involvement of the fovea. This stage appears to be followed by a slowly progressive degeneration of the more peripheral retina spanning the second decade of life. Based on these observations, gene augmentation may lead to local improvements in retinal function in areas with spared photoreceptors, a desirable scenario in clinical trial design. The outlook is much more reassuring than having to demonstrate efficacy as a change of the rate of progression of an otherwise very slow photoreceptor degeneration within the windows of observation of a clinical trial.

### Learnings From Recent IRD Gene Therapy Studies

Following the successful regulatory approval and commercialization of the first retinal gene therapy (voretigene neparvovec-rzyl; LUXTURNA) in 2017, the hope was that gene therapies for other IRDs would rapidly follow. Further ocular gene therapies have, however, yet to be approved by the regulatory agencies.

The choice of endpoints for IRD gene therapy studies is critical and often the subject of intense debate both at a sponsor level and with the various regulatory agencies. Learnings from recent gene-therapy trials for choroideremia to consider when designing IRD gene-therapy clinical trials were shared by Robert MacLaren (University of Oxford, UK). The phase III STAR trial of timrepigene emparvovec (BIIB111/AAV2-REP1) did not meet its primary endpoint of proportion of participants with a greater or equal to 3 line/≥15 Early Treatment Diabetic Retinopathy Study (ETDRS) letter improvement from baseline in best corrected visual acuity (BCVA) at month 12 in the interventional group in comparison to the non-interventional control group.^19^ However, a greater proportion of treated patients did gain ≥10 ETDRS letters or 2 lines of vision compared to the untreated controls. Due to hierarchical statistical methodology, this secondary endpoint did not reach statistical significance. Although a two-line visual acuity gain is a level of improvement deemed clinically significant and appropriate for registration by some regulators, in the absence of a statistically significant primary endpoint, the institutional sponsor suspended the development or timrepigene emparvovec while the complete data set is evaluated.

Learnings from another recent negative outcome of a late-stage clinical trial for IRD were discussed by Bart Leroy (Ghent, Belgium) who presented data from the phase II/III ILLUMINATE clinical trial (ProQR Therapeutics) of sepofarsen RNA therapy in patients with *CEP290*-mediated LCA10.^20^ Although the sepofarsen clinical development program showed significant increases in visual function in phase I/II (post hoc analysis) at 12 months,^21^ in the phase II/III ILLUMINATE study, neither primary nor key secondary endpoints were met, despite positive patient reported outcome measures (PROMs) and empirical observations from the masked clinicians involved in the study. Post hoc analyses conducted by normalizing the measurements acquired from a patient's study eye (active treatment or sham) to their contralateral untreated eye, showed that the efficacy signal seen with sepofarsen across BCVA, FST, and other endpoints including patient reported outcomes (PROs), was more consistent with the results seen in earlier findings, where the contralateral eye was used as the control.^22^

Although there have been examples of post hoc analyses accepted by regulators during the approval process in other therapeutic areas (for example, in Duchenne muscular dystrophy), it was the opinion of some meeting attendees that in IRD these types of analyses would not be deemed acceptable by regulators. In the feedback they received from regulators, ProQR Therapeutics was recommended to run an additional phase II trial, a significant and costly undertaking for a small biotechnology company and the sepofarsen program was subsequently discontinued,^23^ highlighting the current challenges of developing gene therapies for rare conditions. The decision of the sponsors to terminate the program was disappointing for the IRD field, given the number of carefully performed studies that supported not only the safety and efficacy of the intervention, but that also documented the unexpected, prolonged durability of treatment effect.

A reduction in costs associated with gene therapy trials to incentivize biotechnology and pharmaceutical companies to invest in studies that might benefit rare conditions and their associated smaller cohorts of patients was advocated, including the exploration of novel manufacturing batch release phasing to allow an initial cohort of patients to be treated post-approval with clinically manufactured supply. Further commercial batches could be manufactured and Chemistry, Manufacturing and Controls (CMC) approved according to future demand.

A key point in the discussion was made regarding patients who may be treated with gene therapies early versus late in the course of their disease. Enrolling patients for the initial phases of safety-focused gene therapy trials at later disease stages is prudent and generally supported by researchers and regulatory bodies. However, despite demonstrating an appropriate safety profile, unrealistic expectations of acute or subacute efficacy in patients with such later disease stage have conspired against the logical continuation of several studies to target ideal therapeutic windows, which are usually earlier within the degenerative process. One proposed solution was to explore the conditional marketing authorization route as a means to obtain an earlier approval for an intermediate endpoint pending a positive assessment of risk/benefit. In this case, it would be necessary to describe how the surrogate endpoint would be linked to the clinically relevant endpoint and how the comprehensive data would be collected to complete a full marketing authorization. The importance of well-designed phase I studies, and the inclusion of exploratory endpoints to inform later stage studies, rather than as a means of accelerating the development program was emphasized.

### Perspectives From *RDH12* Patients’ Representatives

Patients with *RDH12* and their representatives reported on the unique set of challenges that children and adults affected by these diseases are presented with in their everyday life. The *RDH12* Families and Patients group has now expanded to include over 200 people from over 20 countries, united in their common goal to accelerate the path toward a treatment or even a cure for *RDH12*-associated retinal degenerations. A video capturing the moving testimony of children and adults affected by *RDH12* opened the session dedicated to patients’ perspectives.

Stability of visual function is considered a valuable and clinically meaningful outcome to the patients, both from a physical and mental perspective and its approval would be considered a significant win by the *RDH12* patient community. Even in the later stages of disease, retention of light perception was considered fundamental to patients that wish to retain it as long as possible. From the roundtable discussion following this presentation, it was apparent that there continues to be a clear disconnect in the definition of what constitutes “clinically meaningful” between the *RDH12* patient community and regulators, a not uncommon phenomenon in the rare disease community and one of the reasons the FDA has instituted Patient Advisory meetings.

### Perspectives From the Biotech Industry

Representatives from MeiraGTx and Opus Genetics shared the latest updates on their work in the *RDH12* field. MeiraGTx’s *RDH12* AAV5 gene therapy vector development is now complete, and the vector demonstrates robust biological activity in a mouse model of *RDH12*-associated vision loss. Toxicology studies to support investigational new drug (IND) filing and assays to clear batch release are ongoing. MeiraGTx initiated a natural history study in patients with *RDH12* (target enrollment: *N* = 100 at 7 sites in the United States, United Kingdom, and the European Union) with the goal of following disease progression over time and to correlate phenotypic findings with genotype, however, the discontinuation of this study was recently announced, raising uncertainty around the continuation of their *RDH12* development program.

Opus Genetics also shared the work in progress on their *RDH12* gene therapy program, utilizing an AAV8 photoreceptor targeting vector. The next steps for the near future are to finalize IND filing package and refine the phase I clinical protocol. Opus is sponsoring the *RDH12* arm of the Uni-rare study (NCT05589714), a new natural history study conducted by the Foundation Fighting Blindness (FFB) Clinical Consortium with the aim of characterizing the course of retinal degeneration and vision loss for people with rare genetic mutations.^24^

Both companies had planned to initiate a phase I/II clinical study for safety and tolerability in adults with the hope of enrolling pediatric patients once there is evidence of efficacy. However, recent funding challenges mean that timelines are unclear.

## Roundtable Discussion

When considering future clinical trial design, including outcome measures, there are a number of unique challenges with the *RDH12* population that need to be taken into account, namely severe impairment of retina/vision form early in life (e.g. flat ERG and extremely low if not absent visual acuity since childhood), uncertain expectations of rescue, stabilization or preservation versus improvement/ restoration of vision, heterogeneity of *RDH12* phenotypes (and of IRDs in general) pose significant challenges to predicting treatment benefit, slow expected pace of detectable changes following treatment.

### Clinical Trial Design Considerations

#### Patient Population

The current standard in trials for pediatric disease is to test safety and tolerability in the adult population and exploring signs of efficacy before being able to enroll children in a clinical study. It was acknowledged that these initial adult cohorts are unlikely to benefit from the drug/approach being tested (see above). Although this requirement clearly poses a hurdle to early onset diseases, such as *RDH12*-associated IRDs, it was suggested there may be no need or requirement to prove efficacy in adults if non-clinical animal models can be used to provide such proof; or a single adult individual may be sufficient to provide a valid demonstration of efficacy for a novel drug/therapy. Whether this holds in a clinical trial setting approved by US and EU regulators remains to be seen. Debra Thompson (Kellogg Eye Center, Michigan, USA) reminded the group that part of the necessary non-clinical evidence in mouse models was published by her group in 2019 and showed a protection against light damage susceptibility in mice injected with a gene-therapy vector for *RDH12*-associated IRDs.^25^^,^^26^

#### Clinical Trial Design

The historical need to perform at least two well-controlled trials to show evidence of effectiveness for a new drug was discussed. In rare disease, regulators require that for approval, evidence of effectiveness must now be based on one adequate and well-controlled pivotal clinical study coupled with confirmatory evidence, which can be another trial or another trial site (in the case of voretigene neparvovec-rzyl). Study duration for an *RDH12* trial would be mainly influenced by the measured outcome of choice, with structural outcomes requiring different trial duration than functional, thus underlining the importance of selection of appropriate outcome measures/endpoints tailored to *RDH12*-associated IRDs.

#### Drug Delivery

Areas of the retina exhibiting dissociation between structure and function should be favored for administration of future *RDH12* treatments as opposed to areas that do not show this relationship, as there is a risk that any improvements may not be apparent within the relatively small clinical trial window. Areas that are structurally compromised and devoid of outer segments might benefit from re-activation of the RDH12 enzyme, which would imply the removal of a toxic insult retinaldehyde; this insult will hopefully allow the outer segment re-growth and subsequent restoration of function. Based on clinical trial experience with gene therapies, Daniel Chung (CMO of SparingVision, formerly of Spark Therapeutics) cautioned regarding careful evaluation of the technical aspects of a subretinal injection (i.e. volume and target site). The potential benefits of multiple subretinal injection sites were also considered.

The genotype versus phenotype indication was considered in relation to clinical trial design.

Daniel Chung raised the point that, as for *RPE65*, *RDH12*-associated IRDs have a very variable diagnostic nomenclature. Therefore, the selection of a genetic diagnosis as a clinical trial inclusion criterion (such as “*RDH12*-associated retinal dystrophy”) would be a way to encompass a larger relevant population and would not hinder the design of a clinical trial if parallel precise phenotypic characterization is used to establish eligibility.

### Outcome Measures

#### Optical Coherence Tomography 

Discussion focused on the most relevant OCT parameter as a potential outcome measure for *RDH12*. OCT imaging of the ellipsoid zone (EZ) was proposed, although caution is required due to the presence of sparse areas of structural/functional damage, meaning imaging of the sole EZ area might be less useful than a more inclusive measure encompassing all retinal layers from the surface down to the RPE. This could be beneficial in the selection process of most suitable areas to treat in a situation where the EZ is undetectable. In a scenario of highly compromised retinal structure, such as in *RDH12*, the measurement of the outer nuclear layer (ONL) topography/thickness would be highly influenced by structural alterations in the adjacent layers and a useful approach might be scanning the same area over time to estimate the residual area, as either a percentage or as a volume. A good outcome measure should reflect both the structure and the function of the photoreceptors, which could be achieved by looking at a combination of ONL plus or minus the outer segment length. Measuring the slope of photoreceptor loss over a defined time-period (1–2 years) would be considered a valid outcome measure by some regulators, even in absence of functional change. Other regulators would require evidence that the targeted remaining photoreceptors are indeed functional even in the presence of a reduction of rate of photoreceptor loss, and the clinical relevance of this measure would need to be established.

#### Full Field Light Sensitivity Testing 

Whereas FST has been utilized as a secondary endpoint within prior gene therapy studies,^18^ it is perceived as a valid exploratory tool and not currently deemed acceptable as a primary endpoint by regulatory agencies. This is due in the main to its subjective nature, and for providing an overall rather than more localized measurements of retina sensitivity. The inability of the FST to localize the area of the retina that may show improvement has been argued as a key limitation of FST. Curiously, visual acuity has the exact same limitation, yet it is universally accepted as an outcome measure. There was also concern expressed by the regulators that FST methodology has not been robustly validated. In the opinion of the clinicians, FST was felt to be proven to be reliable and reproducible, with a track record of excellent correlations with conventional measures of vision, such as perimetry. There is also strong correlation of FST with performance of patients in orientation and mobility tests, such as the Multi-Luminance Mobility Test (MLMT), utilized in the pivotal trial for voretigene neparvovec-rzyl, which led to its approval for use in the clinic by the FDA and other international regulatory agencies.^27^^–^^29^ Given the relative preservation of rod function at locations within the peripheral retina of patients with *RDH12*-associated IRD, large changes in retinal sensitivity within the targeted central retina may be required for the classical dark-adapted FST to detect changes.

Other tests, such as microperimetry (MP), may be better suited for FDA approval as a proxy of visual function that can be directly correlated to a clinical and meaningful patient benefit. MP in a predefined area consisting of at least five points and showing the difference of at least seven decibels start to finish, would be an acceptable primary outcome for the FDA. However, from a clinical perspective, there are concerns that the test will be likely out of range and may indeed be unusable in patients with severe early-onset degenerations and unsteady eccentric fixation, such as *RHD12-*associated IRDs, one of the reasons that prompted the development of FST.

#### Mobility Tests

The suitability of the MLMT for patients with *RDH12*-associated IRD was discussed together with adaptations required for the use of mobility mazes as an appropriate endpoint for other IRD patient populations.

Whereas the mean bilateral multi-luminance mobility testing score was the primary endpoint in the pivotal phase III study for voretigene neparvovec-rzyl, there are some utilization challenges associated with the MLMT. The set up can be cumbersome with a relatively large, designated space required. Uniformity of lighting/ luminance levels are difficult to ensure across multiple centers. The administration of the test itself and alteration of maze configuration is time-consuming and only a limited number of tests can be performed in each session. The use of physical objects can constitute a health and safety hazard, plus has the potential for echolocation with some patients. Finally, the videotaping of the tests construes a higher risk of confidentiality breach, requires a reading center and analyses can be complex and lengthy.

To facilitate the functionality and logistics associated with use of mobility mazes/tests, virtual reality (VR) has been deployed.^30^ VR testing confers several advantages over the physical mobility test set up. Once programmed, the equipment is relatively inexpensive, and limited non-designated space is required. Multiple tests can be performed at a variety of luminance levels within 20 minutes, with all quantitative data captured digitally (including timing, gaze direction, acceleration, deceleration, and collisions) and immediately analyzed without the requirement for a reading center. Additionally, unlike the video recordings, there are no personal identifiers. Jean Bennett (UPenn) confirmed that the VR maze being developed at UPenn showed a very close correlation to all standard visual tests (Goldmann Visual Fields, Visual Acuity, FST, etc.) but was not yet directly compared to the MLMT. Regulatory Agencies are accepting of the concept and potential for VR mobility mazes.

#### Patient Reported Outcome Measures 

The potentially severe quality of life (QoL) impact of persons with rare disease and their families and caregivers is well recognized. However, rare diseases are complex, often multi-systemic, chronic, and as most individuals will not be cured within their lifetimes, identifying ways to measure and then improve QoL is critical. PROMs are instruments (questionnaires) designed to capture the status of a patient's health condition that comes directly from the patient, without interpretation of the patient's response by a clinician or anyone else.^31^ Developed using qualitative information (experiences, priorities, and contextual information) from well-defined patient populations, once developed, their validity may not extend to other populations, reinforcing the need for PROMs specifically developed for the IRD patient populations.

There are a number of challenges to be faced when developing PROMs for rare disease,^32^ including phenotypic heterogeneity within and across rare diseases, limited knowledge of natural history, large proportion of children who may not be able to understand questions developed from interviews with adult patients, and the association with progressive and disabling conditions.

PROMs that have been used with the IRD population include the National Eye Institute Visual Function Questionnaire (NEI-VFQ), Michigan Retinal Degeneration Questionnaire (MRDQ), Visual Symptom and Impact Outcomes patient-reported outcomes (ViSIO-PRO), and observer reported outcome (ViSIO-Obs-RO) instruments. NEI-VFQ is ubiquitous but problematic in IRDs due to its multi-dimensionality, lack of documented content validity,^33^ and demonstrated poor fit for *RLBP1*-associated retinitis pigmentosa.^34^ Two such PROMs are the Michigan Retinal Degeneration Questionnaire (MRDQ) developed by the University of Michigan,^35^ and ViSIO-PRO/ViSIO Obs-RO for non-syndromic retinitis pigmentosa and LCA, co-developed by Novartis and Foundation Fighting Blindness.^36^^,^^37^ Although both have been psychometrically validated as well as tested for validity and reliability, of note, only the Obs-RO has been designed for use with children, which could constitute an important advancement in QoL assessment in pediatric *RDH12*-associated IRDs. The importance of mapping any new PROMs tool to the EuroQol-5 Dimension (EQ-5D) was highlighted due to the importance of building a cost-utility analysis for future reimbursement discussions and decisions. Todd Durham (Foundation Fighting Blindness) highlighted the need of collecting information regarding the most common or important symptoms or disease impacts on QoL to design an appropriate PROM strategy to address the requirements to be accepted by regulators and payers.

A recent publication on the use of PROMs in health technology assessments (HTAs) of rare disease treatments,^38^ highlighted the need to use different forms of evidence to understand the QoL impact and to incorporate qualitative insights/data into pivotal regulated studies. Considerations included understanding the impacts of the disease and of treatments, recognizing the nuances in development and administration of PROMS, considering what is feasible and what matters most to the patient population, recognizing that lack of significant effect on a PROM does not imply no QoL benefit, using different forms of evidence to understand QoL impacts, such as patient input, and providing methodological guidance to capture QoL impacts on patients/carers. Relevant outcomes for patients with LCA might be related to performing day-to-day activities, mobility, independence, etc. Mixed-method approaches, which include both qualitative and quantitative methods, to devise and validate new PROMs ad hoc for small, heterogeneous populations common in IRDs should also be considered.

Despite the importance of ensuring the relevance of outcome measures to patients, PROMs in IRDs are still considered as exploratory endpoints in ophthalmology clinical studies by the regulators, despite widely used as outcome measures in neurological clinical trials. Wiley Chambers (FDA) cautioned that none of the PROMs have been approved by the FDA to date as a clinically relevant endpoint for rare diseases. The FDA has issued a step-by-step guidance document for PROM validation and suggest that a performance-based approach might be more amenable to approval rather than a PROM questionnaire. Jane Mosley (European Medicines Agency) added that it is key for PROMs to be validated in an ophthalmology setting, that they be tailored to the population in study and correlated to other visual function measures.

#### Innovative Outcome Measures

There is untapped potential of imaging data in retinal disease through multi-modal image analysis, with discrepancies between functional and structural understandings of *RDH12*-associated retinopathies and the considerable information locked in and underutilized in already acquired retinal images that have functional correlates. Adam Dubis group at UCL and Moorfields Eye Hospital, UK, is developing tools specifically for *RDH12* imaging and is currently in the early stages of collecting imaging data (AF, OCT, and MP) from global partner centers and manually annotating key features relevant to the disease. The second step will include extraction and validation of the features while the third will use semi-supervised deep learning methods to subtype and stage patients with the goal to predict progression and thus facilitate and optimize patient population selection for clinical trials.

#### Light Perception

In response to feedback from individuals with R*DH12-*associated IRDs, and questions from medical experts regarding whether retention of light perception could be considered as a viable outcome measure, Wiley Chambers (FDA) confirmed that retention of perception versus no light perception could already be conceived as an endpoint due to its absolute value (perception versus no perception). However, there is a need to establish a direct correlation between a test and the clinical benefit for the patient. Regulators are open to the development of novel endpoints that directly measure a quantifiable, consistent improvement in the day-to-day activities of the patients.

## Conclusions

This multi-stakeholder collaborative initiative provides a benchmark model for furthering translational research in the field of rare eye diseases. By providing the environment for multiple stakeholders from diverse backgrounds and with differing perspectives to transparently contribute, this event acts as a catalyst for progress and promotes action toward a path for treatments and ultimately a cure for *RDH12*-associated IRDs.

The following were agreed among the group:

• There is a need for robust longitudinal natural history studies to fully elucidate the disease characteristics. Federated, centralized datasets are optimal, ideally leveraging transparent partnerships among academia, patient organizations, and pharmaceutical/biotechnology companies to ensure breadth and leverage operational and financial aspects.
• Outcome measures should be tailored to the specific IRD. The untapped potential within OCT scans should be optimized to further elucidate structural characteristics and their link to functional outcomes/rate of disease progression and how this may vary between individuals, thus facilitating selection of patients for future clinical trials most likely to benefit from gene therapy (or other) approaches.
• The VR MLMT should be tested and validated for use with patients with *RDH12*.
• The use of FST as a primary endpoint should continue to be explored with regulators.
• Work on developing fully validated PROMs should be encouraged in order to capture patient experiences in an age appropriate, quantifiable way to achieve a patient relevant outcome tool acceptable to regulators as a valid endpoint.
• Continued and collaborative engagement with regulatory bodies was encouraged to overcome the challenges posed in clinical trial and outcome measure design in the rare disease *RDH12* patient population, thus providing the best opportunity for future positive, patient-relevant clinical trials, and subsequent regulatory approvals.

## Workshop Participants

Chair:

• Sue Lacey, PhD, Astraea Medical Consulting, UK

Clinicians/Academics:

• Tomas S. Aleman, MD, University of Pennsylvania, USA
• Jean Bennett, MD, PhD, University of Pennsylvania, USA
• Adam Dubis, PhD, University College London, UK
• Bart P. Leroy, MD, PhD, Ghent University, Belgium
• Mariya Moosajee, MBBS, BSc, PhD, FRCOphth, Moorfields Eye Hospital/ UCL, UK
• Debra Thompson, PhD, University of Michigan, USA
• Abigail Fahim, MD, PhD, University of Michigan, USA - virtual
• Robert MacLaren, MD, PhD, University of Oxford, UK - virtual
• Michel Michaelides, MD, PhD, Moorfields Eye Hospital/UCL, UK - virtual
• Mark Pennesi, MD, PhD, Oregon Health & Science University, USA - virtual

Regulators:

• Wiley Chambers, MD, CDER, FDA, USA
• Jennifer Hammer, MD, CBER, FDA, USA
• Ekaterini Tsilou, MD, CBER, FDA, USA
• Jane Moseley, MD, EMA, The Netherlands - virtual

IRD Patient Organizations:

• Courtney Coates, Hope in Focus, USA
• Todd Durham, PhD, Foundation Fighting Blindness, USA
• Jason Menzo, Foundation Fighting Blindness, USA
• Avril Daly, Retina International, Ireland - virtual

RDH12 Alliance Patient Representatives:

• Silvia Cerolini, Eyes on the Future, UK
• Theresa Cole, RDH12 Fund for Sight, USA
• Jogin Desai, MD, India
• Mike Fiore, RDH12 Fund for Sight, USA
• Allison Galloway, RDH12 Fund for Sight, USA
• Lori Kinney, RDH12 Fund for Sight, USA
• Mathew Pletcher, PhD, RDH12 Fund for Sight, USA
• Sylviane DeVel, Candle in the Dark, Belgium - virtual

Industry:

• Dan Chao, MD, PhD, Janssen, USA
• Daniel Chung, MD, PhD, CMO Sparing Vision, USA
• Jennifer Hunt, PhD, Opus GTX, USA
• Shannon Mullican, PhD, Janssen, USA
• Erin O Neil, PhD, Opus GTX, USA
• Sarah Tuller, PhD, Opus GTX, USA
• Ben Yerxa, PhD, Opus GTX, USA
• Robert Zeldin, MD, Meira GTX, UK
• Elin Haf Davies, Aparito, UK - VIRTUAL

Consultants and Partners:

• Madhu Madhusudhan, PhD, LifeArc, UK
• Giorgia Schena, PhD, Science Compass, USA
• Karmen Trzupek, Rare-X, USA
